# Generation of Powerful Human Tolerogenic Dendritic Cells by Lentiviral-Mediated IL-10 Gene Transfer

**DOI:** 10.3389/fimmu.2020.01260

**Published:** 2020-06-30

**Authors:** Michela Comi, Giada Amodio, Laura Passeri, Marta Fortunato, Francesca Romana Santoni de Sio, Grazia Andolfi, Anna Kajaste-Rudnitski, Fabio Russo, Luca Cesana, Silvia Gregori

**Affiliations:** San Raffaele Telethon Institute for Gene Therapy (SR-TIGET), San Raffaele Scientific Institute (IRCCS), Milan, Italy

**Keywords:** dendritic cells, IL-10, cell therapy, immune tolerance, allogeneic transplantation

## Abstract

The prominent role of dendritic cells (DC) in promoting tolerance and the development of methods to generate clinical grade products allowed the clinical application of tolerogenic DC (tolDC)-based therapies for controlling unwanted immune responses. We established an efficient method to generate tolerogenic human DC, producing supra-physiological levels of IL-10, by genetically engineering monocyte-derived DC with a bidirectional Lentiviral Vector (bdLV) encoding for IL-10 and a marker gene. DC^IL−10^ are mature DC, modulate T cell responses, promote T regulatory cells, and are phenotypically and functionally stable upon stimulation. Adoptive transfer of human DC^IL−10^ in a humanized mouse model dampens allogeneic T cell recall responses, while murine DC^IL−10^ delays acute graft-vs.-host disease in mice. Our report outlines an efficient method to transduce human myeloid cells with large-size LV and shows that stable over-expression of IL-10 generates an effective cell product for future clinical applications in the contest of allogeneic transplantation.

## Introduction

Tolerogenic dendritic cells (tolDC) are characterized by low expression of costimulatory molecules, upregulation of inhibitory, and/or modulatory receptors, secretion of low amounts of pro-inflammatory, and high levels of anti-inflammatory cytokines (1). All these factors are responsible for the regulatory capacity of tolDC, which results in the inhibition of effector T cell responses and the induction of T regulatory cells (Tregs) (2). This ability to control immune responses and promote tolerance makes tolDC an interesting candidate for cell therapy approaches in transplantation and in autoimmunity. The seminal study that led to the use of tolDC as inhibitors of allograft rejection stemmed from data demonstrating that adoptive transfer of donor-derived tolDC prolonged heart or pancreatic islet graft survival in mice (3, 4). Since then, several studies in pre-clinical models of transplantation using cells of donor or host origin demonstrated the regulatory capacity of tolDC (1, 5–7). Clinical trials using tolDC to prevent rejection after liver or kidney transplantation (8) (ClinicalTrials.gov identifier: NCT02252055; NCT03726307; NCT03164265), or to restore tolerance in patients with autoimmune diseases, such as rheumatoid arthritis, type 1 diabetes, multiple sclerosis, and Crohn's disease (9–13) have been completed or are ongoing.

TolDC can be differentiated *in vitro* by treatment with immunosuppressive compounds, anti-inflammatory cytokines, or by genetic modifications (14–16). Among the different approaches used for inducing tolDC, IL-10 has been shown to potently modulate the differentiation and functions of myeloid cells (17), leading to the generation of the tolDC with the most powerful tolerogenic characteristics (18).

In the present study, we genetically engineered monocytes prior to DC differentiation with a bidirectional Lentiviral Vector (bdLV) encoding for IL-10 and a marker gene (ΔNGFR). Human myeloid cells are resistant to HIV-1 infection, thus to bdLV transduction. One of the restriction factors mediating this resistance is SAMHD1 (19, 20), which depletes the cytoplasmic pool of deoxynucleoside triphosphates, affecting the reverse transcription process (21). Vpx protein from simian immunodeficiency viruses directs proteasome-mediated degradation of SAMHD1 (22), restoring HIV-1 infection in myeloid cells (19, 20, 23, 24). Therefore, we exploited this natural inhibitor of SAMHD1, treating the monocytes with Vpx-containing viral like particles for 6 h before bdLV transduction (25, 26), and reached up to 98% of transduced monocyte-derived DC. We evaluated the immunotherapeutic role of tolDC generated by bdLV-mediated IL-10 over-expression (DC^IL−10^) in the context of allogeneic tolerance induction. We delineated the phenotype and cytokine profile of DC^IL−10^, we defined their stability upon inflammatory signal exposure, and we analyzed their functionality both *in vitro* and *in vivo*. We also assessed DC^IL−10^ activity *in vivo* and showed that adoptive DC^IL−10^ transfer in humanized mice reduced the allogeneic response in antigen-specific manner, and treatment of allogeneic transplanted mice with DC^IL−10^ autologous to the recipient delayed acute GvHD, prolonging mice survival.

## Materials and Methods

### Vector Production and Titration

VSV-G-pseudotyped third generation bidirectional Lentiviral Vectors (bdLV) were produced by calcium phosphate transfection into 293T cells and concentrated by ultracentrifugation as described previously (27). Titer was estimated by limiting dilution: vector particles were measured by HIV-1 Gag p24 Ag immune capture (NEN Life Science Products, MA, USA), and vector infectivity was calculated as the ratio between titer and total particles. Titers ranged between 5 × 10^8^ and 6 × 10^9^ transducing units/mL, while infectivity between 5 × 10^4^ and 10^5^ transducing units/ng p24. To produce concentrated Vpx-incorporating viral-like particles (VLPs), 293T cells were co-transfected with a VSV-g expressing plasmid and the Simian Immunodeficiency Virus-derived packaging plasmid SIV3+, as previously described (26). For bioluminescence imaging (BLI), luciferase-encoding cDNA was cloned into in LV-GFP instead of the GFP gene and into LV-IL10 instead of ΔNGFR gene to allow *in vivo* tracking of transduced murine DC (DC^NGFR^ and DC^IL−10^, respectively).

### Peripheral Blood Mononuclear Cell (PBMC) Isolation

Human peripheral blood was obtained from healthy donors in accordance with local committee approval (TIGET09), and with the Declaration of Helsinki. Peripheral blood mononuclear cells were isolated by density gradient centrifugation over Lymphoprep™ (Axis-Shield PoC AS, Norway).

### Human Dendritic Cells

CD14^+^ cells were isolated from PBMC by positive selection using CD14 MicroBeads (Miltenyi Biotech, Germany) according to the manufacturer's instructions. Cells were cultured in RPMI 1640 (Lonza, Switzerland) with 10% fetal bovine serum (FBS) (Euroclone, Italy), 100 U/ml penicillin/streptomycin (Lonza, Switzerland), 2 mM L-glutamine (Lonza, Switzerland), at 10^6^ cells/ml in a 1 ml volume in a 24-well culture plate, supplemented with rhGM-CSF (Miltenyi Biotech, Germany) at 100 ng/ml and rhIL-4 (Miltenyi Biotech, Germany) at 10 ng/ml for 7 days at 37°C with 5% CO_2_. One ml per well of fresh pre-warmed medium with cytokines, at final concentration as above, was added on day 3. To obtain mature DC (mDC), un-transduced DC were activated at day 5 with 1 μg/ml of LPS (Sigma Aldrich, CA, USA). For DC transduction, monocytes were exposed for 6 h to Vpx-VLP and then were transduced with the indicated vectors at Multiplicity of Infection (MOI) of 5 at day 0, 2, or 5. After overnight incubation, half of the medium was replaced with fresh medium supplemented with cytokines to dilute the vector concentration. For DC^IL−10^ generation, 10 ng/ml of rhIL-10 (CellGenix, Germany) was added at day 0. In some experiments, DC^IL−10^ were activated at day 6 with 1 μg/ml of LPS (Sigma Aldrich, CA, USA) or with 10 μg/ml of Poli (I:C) (InvivoGen, CA, USA). DC were harvested on day 7 for phenotypical, molecular, and functional analyses.

In some experiments, 10^5^ DC were plated in 200 μl of final volume, alone or in the presence of the following stimulation: 1 μg/ml of LPS (Sigma Aldrich, CA, USA), 10^8^ cells/ml of Heat Killed Listeria Monocytogenes, 1 μg/ml of Flagellin *S. typhimurium*, 10 μg/ml of Poli (I:C), 5 μM of ODN2006 (CpG) (InvivoGen, CA, USA) or a mix of 10 ng/ml for each cytokine of IL-1β, TNF-α, and IL-6 (R&D Systems, MN, USA). After 24 h, supernatants were collected to evaluate the cytokine secretion profile by ELISA, and cells were analyzed by flow cytometry.

### Human T Cells

CD3^+^, CD4^+^, and CD8^+^ T cells were purified from PBMC by negative selection using their respective human T cell Isolation kit (Miltenyi Biotech, Germany) according to the manufacturer's instructions. All T cell cultures were performed in X-VIVO 15 medium (Lonza, Switzerland), supplemented with 5% human serum (Sigma Aldrich, CA, USA), and 100 U/ml penicillin/streptomycin (Lonza, Switzerland). T cells were labeled with Cell Proliferation Dye eFluor^®^ 670 (eBioscience, CA, USA) according to manufacturer's instructions and stimulated with 10^4^ allogeneic DC (10:1, T:DC). After 5 days, T cells were collected, washed, and their phenotype and proliferation were analyzed by flow cytometry.

For T cell differentiation, 10^6^ CD4^+^ T cells were cultured with 10^5^ allogeneic DC (10:1, T:DC). After 10 days, primed T cells were collected and purified using CD4 Microbeads (Miltenyi Biotech, Germany). T cells stimulated with DC^UT^ are referred to as T(DC^UT^) cells, while those stimulated with DC^GFP^ as T(DC^GFP^) cells. T cells cultured with unstimulated DC^IL−10^ are referred to as T(DC^IL−10^) cells, while those cultured with LPS- or Poli I:C-stimulated DC^IL−10^ are referred to as T(DC^IL−10−LPS^) or T(DC^IL−10−POLI^) cells, respectively.

For recall response proliferation, primed CD4^+^ T cells were stained with Cell Proliferation Dye eFluor^®^ 670 (eBioscience, CA, USA) and plated with DC^UT^ from the same donor used for priming (10:1, T:DC). After 3 days of stimulation, T cells were collected, washed, and proliferation was evaluated by flow cytometry.

To evaluate the suppressive activity of T(DC^IL−10^), T(DC^IL−10−LPS^), or T(DC^IL−10−POLI^) cells, we stained total CD4^+^ T cells (responder cells) autologous to T cells used in priming with Cell Proliferation Dye eFluor^®^ 450 (eBioscience, CA, USA), and activated them with mDC from the same donor used for priming. T(DC^IL−10^), T(DC^IL−10−LPS^), or T(DC^IL−10−POLI^) cells stained with Cell Proliferation Dye eFluor^®^ 670 (eBioscience, CA, USA), were added at a 1:1 ratio with responder cells (total T:DC ratio is 10:1). After 4 days, the percentages of divided responder T cells were calculated by proliferation dye dilution by flow cytometer.

### Cytokine Determination

For DC, the indicated number of cells were plated in 200 μl of final volume and left unstimulated or activated with 200 ng/ml of LPS (Sigma, CA, USA) and 50 ng/ml of IFN-γ (R&D System, MN, USA). Supernatants were collected after 48 h and levels of IL-6, IL-10, IL-12, and TNF-α were tested.

For CD4^+^ T cells, IL-10, and IFN-γ production was quantified in co-culture supernatants. Cytokine concentration was evaluated by standard sandwich ELISA, with purified and biotinylated antibody couples (Becton Dickinson, CA, USA).

### Mice

NSG, Balb/c and C57Bl/6 female mice were purchased from Charles-River Italia. All mice were fed standard laboratory diet and maintained under standard laboratory conditions free of specific pathogens. All animal care procedures were performed according to protocols approved by the OSR Institutional Animal Care and Use Committee (IACUC protocol #488, #632, and #748), following the 3R principles (replacement, reduction, and refinement) and the Decreto Legislativo #116 dated January 27th, 1992, from the Italian Parliament.

### Modulation of Immune Response in Humanized Mice

Two–five days old NSG (NOD.Cg-PrkDC^cid^ Il2rg^tm1^WjI/SzJ, JAX mouse strain) mice were sub-lethally irradiated (1.5cGy) and injected intra-hepatically 5–7 h later with 10^5^ CD34^+^ (purity ≥ 95%, Lonza), as previously described (28). Percentages of human cells in peripheral blood were monitored by flow cytometry starting from 8 weeks post-transplant. Once human engraftment was stable and T cell repopulation detectable (usually around 11–13 weeks post-transplant), humanized mice (huMice) were immunized by intravenous injection (i.v.) of 5 × 10^6^ irradiated allogeneic CD3^−^ cells (6,000 rad), magnetically isolated with Dynabeads CD3 (Thermo Fisher Scientific, MA, USA) from human PBMC. One week later, human T cell percentages were assessed by flow cytometry, huMice were randomly assigned to experimental groups and injected with 3 × 10^5^ un-transduced DC, or 3 × 10^5^ un-transduced plus 3 × 10^5^ transduced DC, differentiated from CD14^+^ monocytes isolated from the same donor used for CD3^−^ purification. After peripheral blood phenotyping at day 10 and 13, huMice were suppressed at day 13 and peripheral blood harvested.

### Murine DC Differentiation and Transduction

Female Balb/c mice were sacrificed and bone marrow (BM) was harvested. BM cells were cultured in IMDM (Lonza, Switzerland) with 10% fetal bovine serum (FBS) (Euroclone, Italy), 100 U/ml penicillin/streptomycin (Lonza, Switzerland), 2 mM L-glutamine (Lonza, Switzerland), at 10^6^ cells/ml in a 1 ml volume in a 24-well culture plate, supplemented with rmGM-CSF (R&D Systems, MN, USA) at 25 ng/ml for 9 days at 37°C with 5% CO_2_. One ml per well of fresh pre-warmed medium with rmGM-CSF, at final concentration as above, was added on day 3 and, after removal of 1 ml of supernatant from the culture, at day 5. DC were activated at day 7 with 200 ng/ml of LPS (Sigma Aldrich, CA, USA). For DC transduction, cells were transduced with indicated vectors at MOI of 10 at day 2.

### Splenocyte Stimulation

Spleen mononuclear cells were isolated from female C57Bl/6 (H-2b) mice and stained with Proliferation Dye eFluor^®^ 670 (eBioscience, CA, USA). 10^5^ splenocytes were plated with 10^4^ DC^GFP^ or DC^IL−10^ differentiated from BM of female Balb/c mice and collected after 5 days to assess the proliferation by dye dilution.

### Bioluminescence Image Acquisition and Analysis

Female Balb/c (H-2d) mice were lethally irradiated (10 Gy) and intravenously injected with 2 × 10^6^ DC^NGFR^ or DC^IL−10^, with or without the addition of 10^7^ Balb/c (H-2d) BM cells. Small-animal bioluminescence imaging (BLI) was performed using the IVIS Spectrum CT System (Perkin Elmer). The system is composed of a low-noise, back-thinned, back-illuminated charge-coupled device (CCD) camera cooled at −90°C with a quantum efficiency in the visible range above 85%. Each mouse received an intravenous injection of 150 mg luciferin/kg body weight 10 min before BLI. During image acquisition, the animals were kept at 37°C and under gaseous anesthesia (2–3% isoflurane and 1 L/min oxygen). Dynamic BLI was performed by acquiring a set of images every 2 min from 10 to 20 min after luciferin injection to detect the highest BLI signal. The images were obtained using the following settings: exposure time = auto, binning = 8, and field of view = 23.4 cm. Dark images were acquired before and then subtracted to bioluminescence images; no emission filters were used during BLI acquisitions. BLI image analysis was performed by placing a region of interest (ROI) over the body of the mouse (tail excluded) and by measuring the total flux (photons/seconds) within the ROI. Images were acquired and analyzed using Living Image 4.5 (Perkin Elmer).

### *In vivo* Acute Graft vs. Host Disease Model

Acute graft vs. host disease (GvHD) was induced by a single intravenous injection of BM cells (10 × 10^6^) supplemented with 5 × 10^6^ spleen mononuclear cells isolated from female C57Bl/6 (H-2b) mice into recipient female Balb/c (H-2d) mice lethally irradiated (10 Gy total body irradiation). Recipients received single intravenous injections of host-matched DC^GFP^ or DC^IL−10^ (2 × 10^6^) 3 days after transplantation. Recipients were monitored once every other day from the day of transplantation to determine survival time, body weight and score (fur, hunch, skin lesion, mobility). Moribund mice were euthanized for ethical reason (more than 25% of weight loss or score higher than six).

### Flow Cytometry

Fluorochrome-conjugated antibodies against the following antigens were used for human DC staining: NGFR, CD1a, CD14, CD83, CD86, HLA-DR, CD16, CD163 (Becton Dickinson, CA, USA), and CD141 (Miltenyi Biotech, Germany), HLA-G (Exbio, Czech Republic), ILT4 (Beckman Counter, NJ, USA). The following fluorochrome-conjugated antibodies were used for human CD3^+^ T cell staining: anti-CD3, anti-CD4, anti-CD8, and anti-CD45RA (Becton Dickinson, CA, USA), anti-CD49b and anti-LAG-3 (Miltenyi Biotec, Germany). For Tr1 cell detection, CD4^+^ T cells were stained as previously described (29).

Fluorochrome-conjugated antibodies against the following antigens were used for murine DC staining: NGFR, CD11c, CD80, CD83, CD86, I-A/I-E (Becton Dickinson, CA, USA). The following fluorochrome-conjugated antibodies were used for murine splenocyte staining: anti-CD3, anti-CD4, and anti-CD8 (Becton Dickinson, CA, USA).

FcR Blocking Reagent (Miltenyi Biotech, Germany for human sample and Becton Dickinson, CA, USA for mice samples) was used in all preparations to avoid non-specific staining. Briefly, cells were centrifuged and re-suspended in Dulbecco's Phosphate-Buffered Saline (DPBS, Corning) supplemented with 2% FBS (Lonza, Switzerland). Cells were incubated at room temperature for 15 min, centrifuged and fixed with 1% formaldehyde solution methanol-free (Thermo Fisher Scientific, MA, USA). For cultured cells, the described passages where preceded by staining with LIVE/DEAD™ Fixable Dead Cell Stain Kit (Invitrogen, CA, USA) following manufacturer's instructions.

For huMice blood staining, 100 μl of whole blood was stained with antibodies against surface markers for 15 min at room temperature. Cells were then fixed, permeabilized and stained with anti-KI67 (Becton Dickinson, CA, USA) using Foxp3/Transcription Factor Staining Buffer Set (eBioscience, USA). Samples were acquired using the FACSCanto II or Fortessa Flow Cytometers (Becton Dickinson, CA, USA) and data were analyzed with FlowJo software (FlowJo LLC, USA).

### Statistical Analysis

Wilcoxon matched pairs test (two-tailed) were used for statistical analysis. All results are presented as mean values ± standard deviation, unless differently specified in the figure legend. Differences were regarded as significant at ^*^P ≤ 0.05, ^**^P ≤ 0.01, and ^***^P ≤ 0.001. Results were analyzed using GraphPad Prism 5.0 (GraphPad Software, CA, USA).

## Results

### Monocytes Can Be Stably and Highly Transduced With Bidirectional Lentiviral Vectors

**Figure 1:**
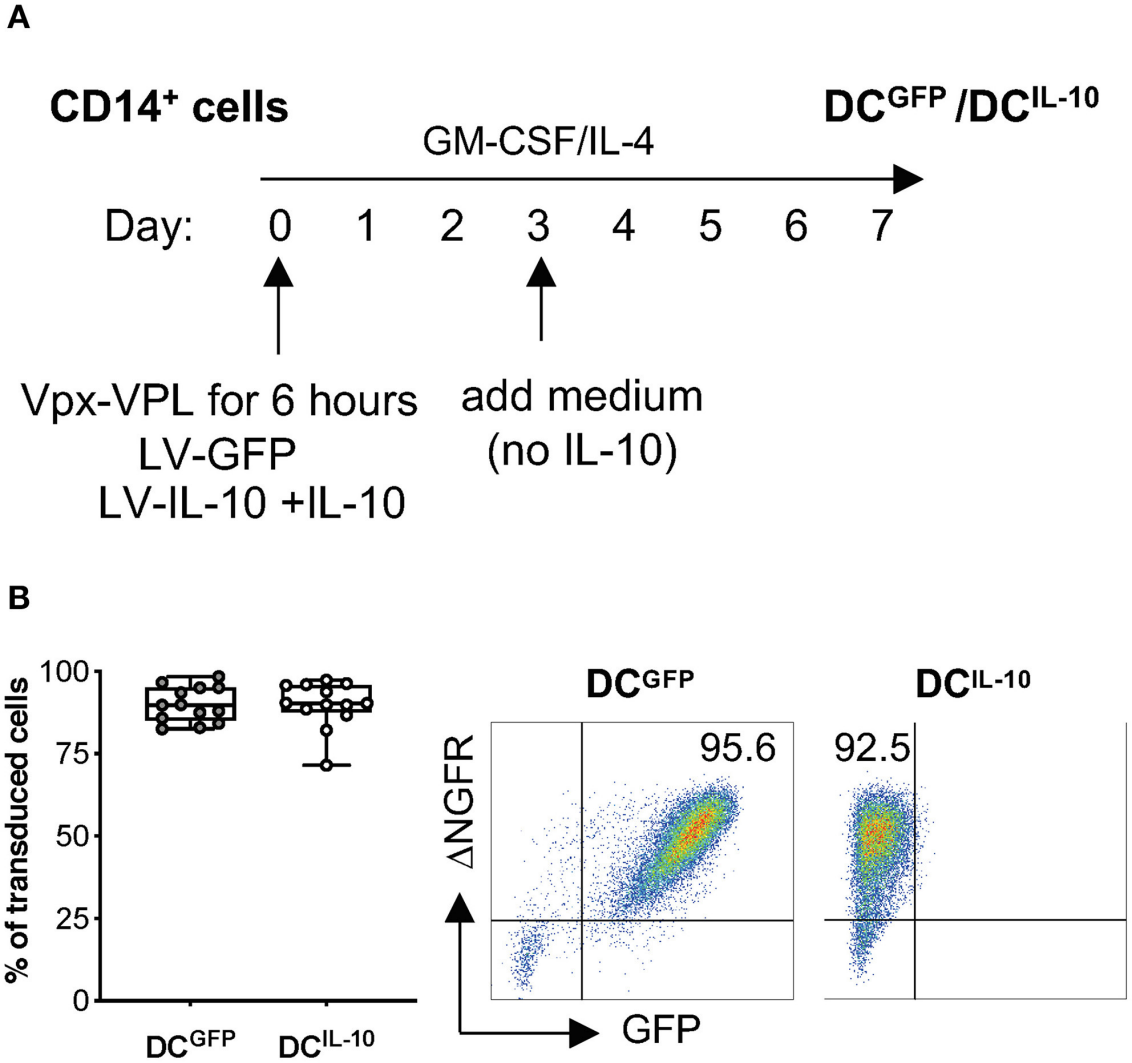
BdLV-mediated gene transfer to engineer human monocyte-derived DC. CD14^+^ cells isolated from peripheral blood of healthy subjects were treated with Vpx-VLP for 6 h and then transduced with LV-IL-10 (DC^IL−10^) in the presence of exogenous IL-10 at day 0 during DC differentiation. As control, DC transduced with LV-GFP (DC^GFP^) were differentiated from the same donors. **(A)** Protocol for bdLV-mediated transduction of monocyte-derived DC is depicted. **(B)** Transduction efficiency was quantified based on ΔNGFR expression on differentiated DC. Each dot represents a single donor (*n* = 13), lines indicate median, while whiskers are minimum and maximum level (left panel). Dot plots from one representative donor are presented. Numbers represent percentage of positive cells (right panel).

Lentiviral vector (LV) transduction of monocyte-derived DC has been described; however, transduction efficiency is generally low due to the inhibition of reverse transcription in human myeloid cells mediated by SAMHD1 (19, 20, 23). The use of polybrene in combination with simian immunodeficiency virus (SIV)-derived accessory protein Vpx during LV transduction has been proposed to overcome this limitation (26, 30). We optimized the transduction protocol of human monocyte-derived DC with LV encoding large size plasmid (~10 kb), by pre-treating CD14^+^ cells with viral-like particles containing Vpx (Vpx-VLP) in the absence of polybrene before exposure to a bdLV co-encoding for GFP and ΔNGFR (LV-GFP). CD14^+^ cells were pre-treated or not with Vpx-VLP for 6 h on day 0, 2, or 5 before LV-GFP exposure during monocyte-derived DC differentiation, and transduction efficiency was evaluated at the end of differentiation (Figure S1A). Pre-treatment with Vpx-VLP at all time points analyzed improved transduction, reaching the highest efficiency when cells were pre-treated with Vpx-VLP at day 0 (up to 95% of ΔNGFR^+^ DC, Figure S1B). As expected, in the absence of Vpx-VLP, DC were transduced at very low levels (<6% of ΔNGFR^+^ DC), irrespectively of the day of transduction (Figure S1B). We then applied the above protocol to transduce CD14^+^ cells with a bdLV co-encoding for IL-10 and ΔNGFR (LV-IL-10) (31). Although the transduction efficiency of DC^IL−10^ was similar to that of DC^GFP^ (not shown), the differentiated population contained two distinct cell subtypes (CD14^−^CD16^−^ and CD14^+^CD16^+^; Figure S1C). Since the presence of IL-10 from day 0 of differentiation results in high CD14 and CD16 expression in DC-10 (32), we hypothesized that the observed heterogenicity in DC^IL−10^ was due to a later exposure to IL-10. For this reason, we added exogenous IL-10 at day 0 during CD14^+^ cell pre-treatment with Vpx-VLP and exposure to LV-IL-10 (Figure 1A) and we obtained a homogenous population of CD14^+^CD16^+^ DC^IL−10^ (Figure S1C), with an average transduction efficiency of 90% for DC^IL−10^, which was superimposable to that of DC^GFP^ (Figure 1B). In conclusion, we established an efficient protocol to transduce human monocytes during DC differentiation with large-size LV.

### Human DC^IL-10^ Express DC-10-Associated Markers and Secrete Supra-Physiological Levels of IL-10

**Figure 2:**
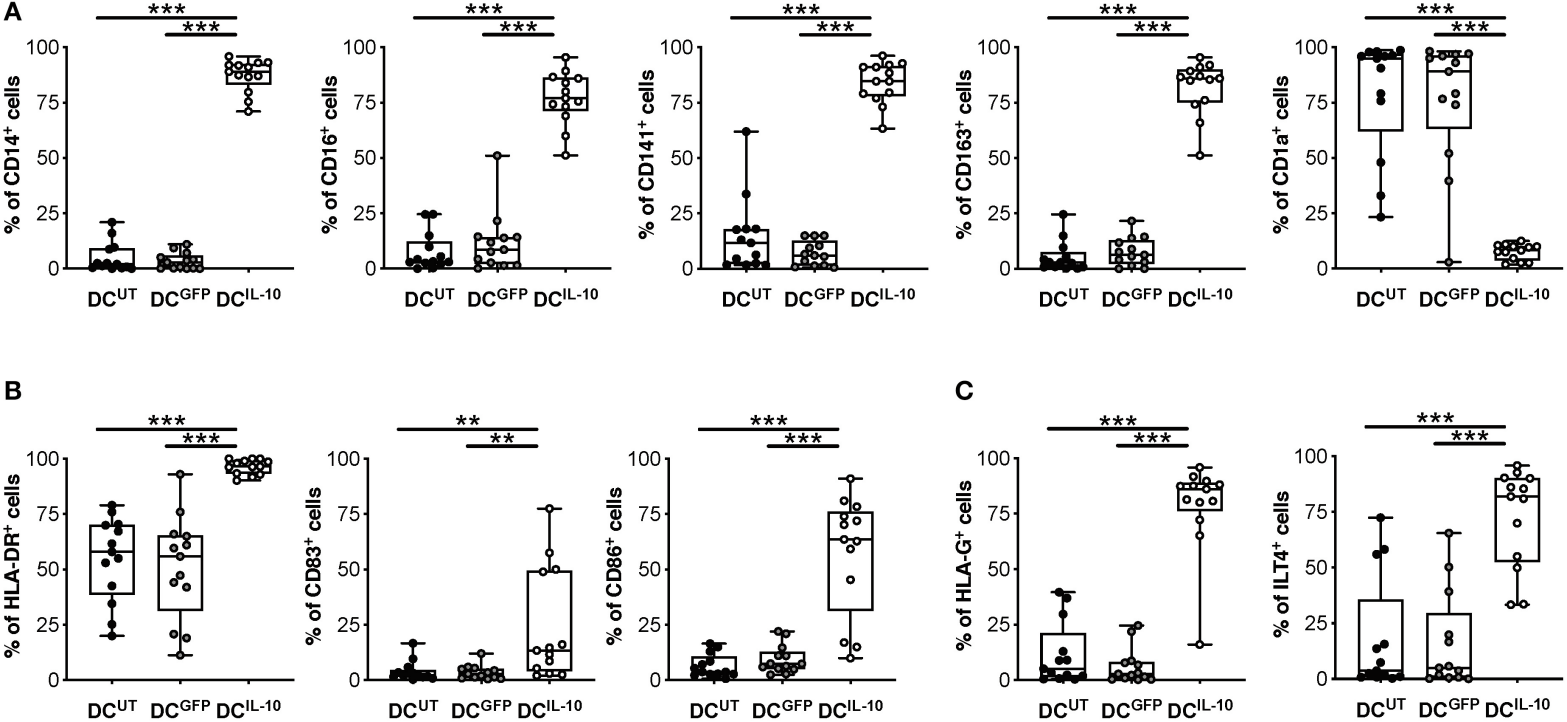
DC^IL−10^ are myeloid mature cells and express markers associated with tolerogenic DC-10. DC^IL−10^ were differentiated as described in method section. At the end of differentiation, the expression of the indicated markers was evaluated by flow cytometry. **(A)** Markers associated with DC-10 differentiation (CD14, CD16, CD163, CD141 CD1a); **(B)** HLA class II (HLA-DR), and co-stimulatory (CD83 and CD86) molecules; **(C)** Tolerogenic molecules (HLA-G and ILT4). Each dot represents a single donor (*n* = 13), lines indicate median, while whiskers are minimum and maximum levels. ^**^P ≤ 0.01, ^***^P ≤ 0.001 (Wilcoxon matched pairs test, two-tailed).

We previously described a subset of human DC, called DC-10, that are differentiated *in vitro* through exposure of monocytes to IL-10 during DC differentiation (32) and, recently, we identified DC-10-specific biomarkers (33). Since DC^IL−10^ are differentiated in the presence of IL-10, it was not surprising that they expressed the DC-10 markers CD14, CD16, CD141, and CD163 at significantly higher levels compared to un-transduced (DC^UT^) and LV-GFP transduced (DC^GFP^) cells, while the levels of CD1a, marker of the classical monocyte-derived DC, were significantly lower (Figure 2A). We next evaluated the expression of MHC class II and costimulatory molecules, important for appropriate antigen presentation to and activation of T cells. In all donors tested, HLA-DR expression in DC^IL−10^ was significantly higher than in DC^UT^ and DC^GFP^, while the expression of CD83 and CD86 on DC^IL−10^ was variable among donors tested, with some donors showing higher and others similar expression levels compare to control DC (Figure 2B). The levels of the tolerogenic molecules HLA-G and ILT4, known to be required for T regulatory type 1 (Tr1) cell induction *via* DC-10 (32), were significantly higher on DC^IL−10^ compared to control DC (Figure 2C).

**Figure 3:**
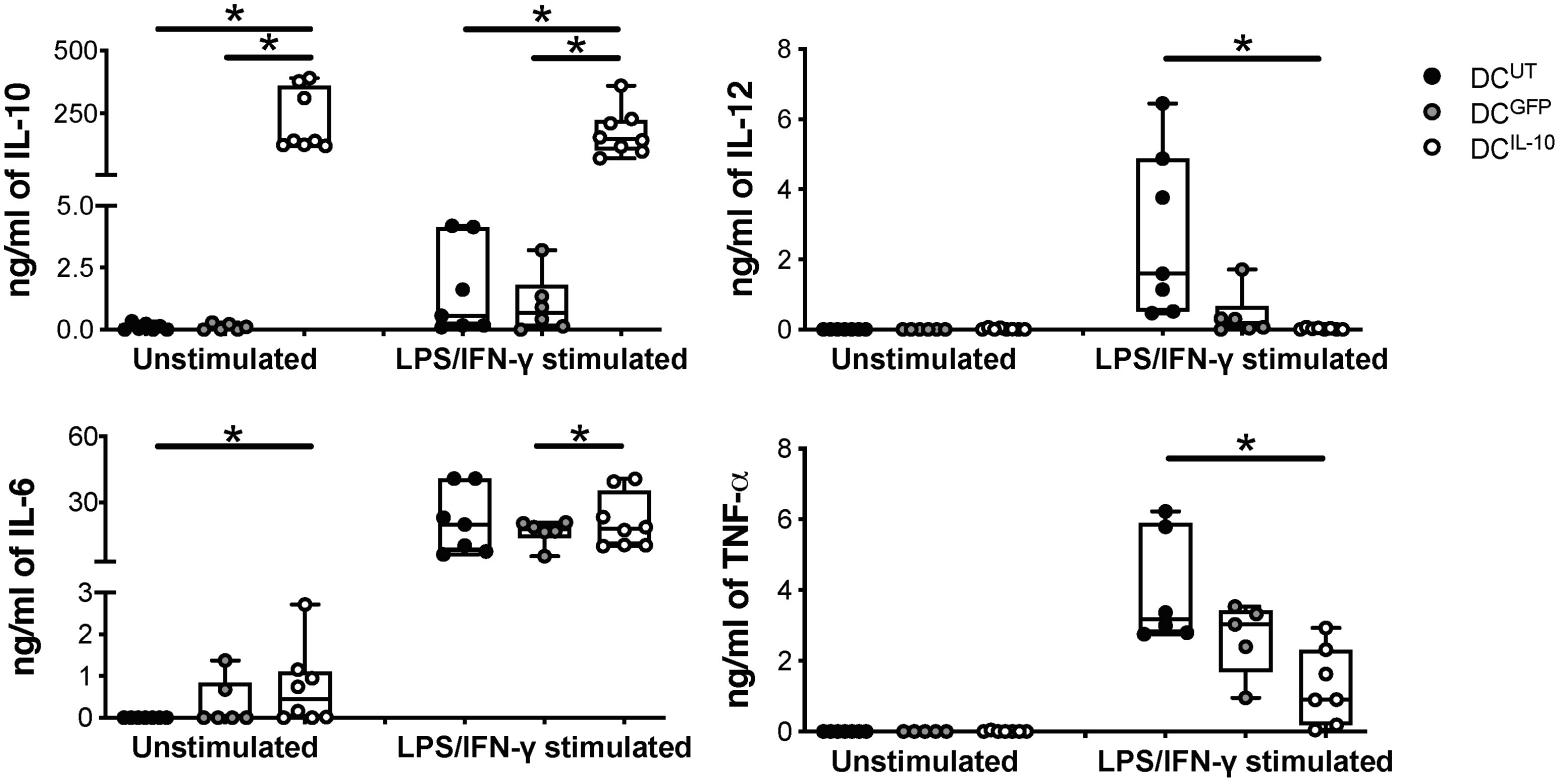
DC^IL−10^ secrete supra-physiological levels of IL-10 and low levels of pro-inflammatory cytokines at steady state and upon activation. At the end of differentiation, 2 × 10^5^ DC were plated in 200 μl and left unstimulated or stimulated with LPS and IFN-γ for 48 h. Concentration levels of IL-10, IL-12, IL-6, and TNF-α in culture supernatants were evaluated by ELISA. Each dot represents a single donor (*n* = 6–8), lines indicate median, while whiskers are minimum and maximum levels. ^*^P ≤ 0.05 (Wilcoxon matched pairs test, two-tailed).

As expected, DC^IL−10^ secreted high amounts of IL-10 at steady state and upon LPS/IFN-γ stimulation, in the absence of IL-12. Conversely, stimulated DC^UT^ and DC^GFP^ produced high levels of IL-12 and variable levels of IL-10, always lower compared to that of DC^IL−10^. There were no differences in the secretion of IL-6 upon stimulation, while higher IL-6 secretion in DC^IL−10^ compared to DC^UT^ at steady state was detected. TNF-α release was not observed in the absence of stimulation, whereas the lowest levels of TNF-α were measured in stimulated DC^IL−10^ compared to controls (Figure 3). Overall, DC^IL−10^ showed a high IL-10/TNF-α and IL-10/IL-12 ratio indicative of their skewing toward tolerance, opposite to the pro-inflammatory cytokine profile displayed by DC^UT^ and DC^GFP^. Altogether, our results show that stable and enforced expression of IL-10 in human monocyte-derived DC through bdLV-mediated gene transfer promotes the differentiation of mature tolerogenic DC-10-like cells, which express HLA-G and ILT4.

### Human DC^IL-10^ Modulate Allogeneic T Cell Responses and Promote Allo-Specific Tr1 Cells *in vitro*

**Figure 4:**
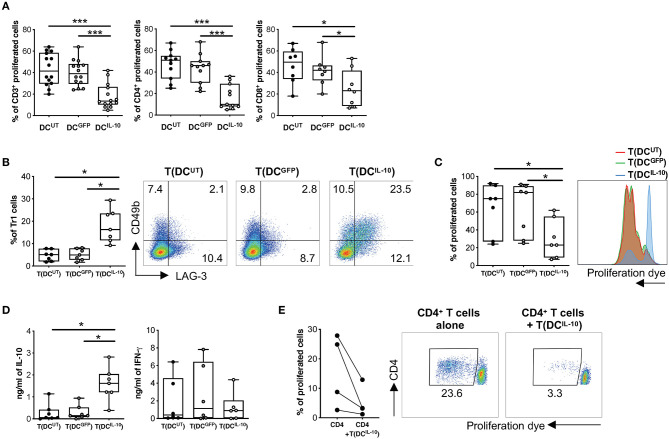
DC^IL−10^ promote Tr1 cell differentiation *in vitro*. **(A)** Differentiated DC were used to stimulate at 10:1 ratio allogeneic CD3^+^, CD4^+^, and CD8^+^ T cells isolated from peripheral blood of healthy subjects and stained with proliferation dye. Proliferation was assessed by dye dilution after 5 days. Each dot represents a single donor (*n* = 14), lines indicate median, while whiskers are minimum and maximum levels. **(B)** Allogeneic CD4^+^ T cells were isolated from peripheral blood of healthy subjects and stimulated with DC at 10:1 ratio for 10 days. After culture, T cells cultured with DC^UT^ [T(DC^UT^)], with DC^GFP^ [T(DC^GFP^)] or with DC^IL−10^ [T(DC^IL−10^)] were collected and the frequency of CD49b^+^LAG-3^+^ Tr1 was evaluated by flow cytometry. Each dot represents a single donor (*n* = 7), lines indicate median, while whiskers are minimum and maximum levels (left panel). Dot plots from one representative donor are shown. Percentages of positive cells are indicated (right panel). **(C,D)** After culture, T(DC^UT^), T(DC^GFP^), and T(DC^IL−10^) cells were purified by positive selection and stained with a proliferation dye prior to re-stimulation with mDC, differentiated from the same donor used in primary stimulation. At day 3 proliferation was evaluated by flow cytometry (*n* = 7). Percentage of proliferated cells in the precursor population was calculated (left panel) and histograms from one representative donor are shown (right panel) **(C)**. IL-10 and IFN-γ in cell culture supernatants was evaluated by ELISA (*n* = 7) **(D)**. Each dot represents a single donor, lines indicate median, while whiskers are minimum and maximum levels. ^*^P ≤ 0.05, (Wilcoxon matched pairs test, two-tailed). **(E)** CD4^+^ T cells autologous to CD4^+^ cells used in primary stimulation were stimulated with mDC, differentiated from the same donor used in primary stimulation, in presence or absence of T(DC^IL−10^) cells at 1:1 ratio. Percentage of proliferated cells at the end of the culture was calculated by overall proliferation dye dilution. Each dot represents a single donor (*n* = 4), lines indicate median, while whiskers are minimum and maximum levels. ^*^P ≤ 0.05 (Wilcoxon matched pairs test, two-tailed) (left panel). Dot plots from one representative donor are shown and percentages of gated cells are indicated (right panel). ^*^P ≤ 0.05, ^***^P ≤ 0.001 (Wilcoxon matched pairs test, two-tailed).

We then proceeded to the functional characterization of DC^IL−10^ and found that they induced significantly lower proliferative responses in allogeneic CD3^+^ T cells when compared to that elicited by control DC^UT^ and DC^GFP^, in both CD4^+^ and CD8^+^ T cells (Figure 4A). According to their similarity to DC-10 and their ability to secrete high levels of IL-10, stimulation of allogeneic CD4^+^ T cells with DC^IL−10^ allowed the differentiation of cells—T(DC^IL−10^) cells—that contained a significantly higher proportion of Tr1 cells compared to T cells primed with DC^UT^ and DC^GFP^—T(DC^UT^) and T(DC^GFP^) cells, respectively—(Figure 4B). In line with the presence of Tr1 cells, T(DC^IL−10^) cells re-stimulated with mature DC (mDC), autologous to DC used for priming, were hypo-responsive (Figure 4C), and produced significantly higher level of IL-10, but similar levels of IFN-γ compared to both T(DC^UT^) and T(DC^GFP^) cells (Figure 4D). Notably, anergy was alloantigen-specific, since the stimulation with a third party mDC induced comparable levels of proliferation in all primed T cells (Figure S2). Moreover, T(DC^IL−10^) cells suppressed the proliferation of autologous CD4^+^ T cells stimulated with mDC from the same donor used for priming, with an average of 67% of suppression (Figure 4E). Overall, these findings indicate that bdLV-mediated IL-10 gene transfer in DC induces a cell population endowed with the ability to modulate allogeneic T cell responses and promote the differentiation of alloantigen-specific Tr1 cells *in vitro*.

### Human DC^IL-10^ Are Phenotypically and Functionally Stable Cells

**Figure 5:**
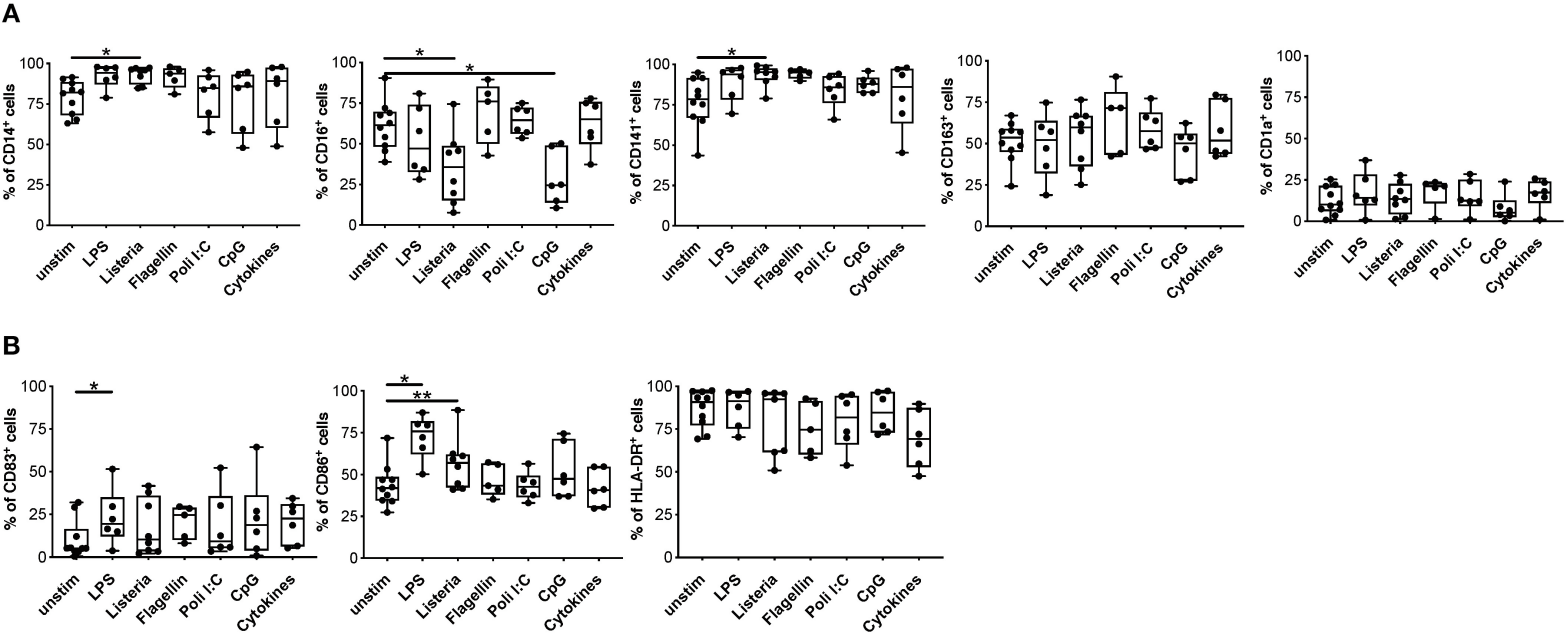
Upon activation, DC^IL−10^ maintain their phenotype. At the end of differentiation, DC^IL−10^ were activated with LPS, Heat Killed Listeria Monocytogenes, Flagellin S. typhimurium, Poli I:C, ODN2006 (CpG), or a mix of cytokines (IL-1β, TNF-α, and IL-6). After 24 h, the expression of the indicated markers was evaluated by flow cytometry. **(A)** Markers associated with DC-10 differentiation (CD14, CD16, CD163, CD141 CD1a); **(B)** HLA class II (HLA-DR), and co-stimulatory (CD83 and CD86) molecules (*n* = 5–10). Each dot represents a single donor, lines indicate median, while whiskers are minimum and maximum levels. ^*^P ≤ 0.05, ^**^P ≤ 0.01 (Wilcoxon matched pairs test, two-tailed).

One of the major hurdles in the use of tolDC as cell product is their stability, thus we investigated the impact of a pro-inflammatory milieu on the phenotype and functions of DC^IL−10^. To this end, we assessed phenotype and function of DC^IL−10^ stimulated *in vitro* with different toll-like receptor (TLR) agonists (LPS, Listeria, Flagellin, Poli I:C, and CpG) or with a mix of pro-inflammatory cytokines (IL-1β, TNF-α, and IL-6). No major changes in DC^IL−10^ phenotype were observed upon activation, with the exception of a significant down-regulation of CD16 upon Listeria and CpG stimulation, and a significant upregulation of CD86 upon Listeria- and LPS-mediated activation (Figures 5A,B). In line with results obtained in DC-10 (33), DC^IL−10^ stably expressed CD141, and CD163 independently from the stimuli used (Figure 5A). Furthermore, DC^IL−10^ maintained their ability to secrete high amounts of IL-10 in the absence of IL-12, independently from the stimulation used. Activation with LPS, Listeria, and Flagellin of DC^IL−10^ promoted significantly higher levels of IL-6 and TNF-α compared to unstimulated conditions (Figure S3). Thus, DC^IL−10^ are phenotypically stable cells and activation with some TLR agonists further promote their activation, as demonstrated by the up-regulation of CD86 and the increase in IL-6 and TNF-α secretion.

**Figure 6:**
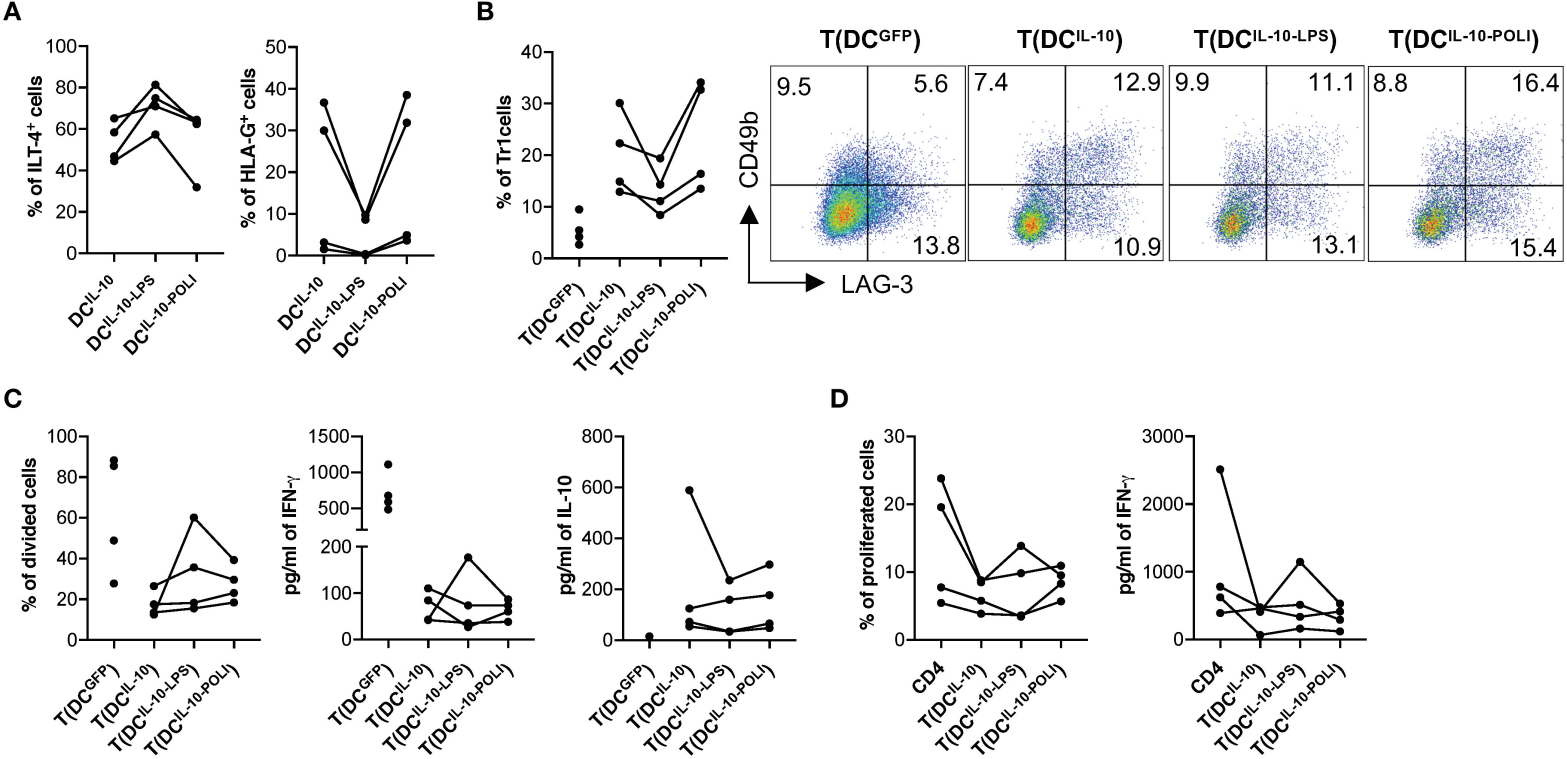
DC^IL−10^ are functionally stable cells. **(A)** At the end of differentiation, DC^IL−10^ were activated with LPS or Poli I:C for 24 h. HLA-G and ILT4 expression was assessed by flow cytometry. Each dot represents a single donor (*n* = 4). **(B)** On day 5 during DC differentiation, DC^IL−10^ were activate with LPS (DC^IL−10−LPS^), or Poli I:C (DC^IL−10−POLI^), or left unstimulated (DC^IL−10^). At the end of the differentiation, DC were used to stimulate allogeneic CD4^+^ T cells, cultured at 10:1 T:DC ratio. After 10 days, CD4^+^ T cultured with DC^IL−10^ [T(DC^IL−10^)], with DC^IL−10−LPS^ [T(DC^IL−10−LPS^)], with DC^IL−10−POLI^ [T(DC^IL−10−POLI^)], or with DC^GFP^ [T(DC^GFP^)] were collected and the frequency of CD49b^+^LAG-3^+^ Tr1 cells was evaluated (*n* = 4) by flow cytometry. Each dot represents a single donor. Dot plots from one representative donor are shown and percentages of positive cells are indicated (right panel). **(C)** After culture, T(DC^IL−10^), T(DC^IL−10−LPS^), T(DC^IL−10−POLI^), and T(DC^GFP^) were purified by positive selection and stained with proliferation dye prior to re-stimulation with mDC, differentiated from the same donor used in primary stimulation. After 3 days, proliferation was evaluated by flow cytometry and the percentage of proliferated cells in the precursor population was calculated (left panel). IFN-γ and IL-10 production in culture supernatants was evaluated by ELISA (central and right panels, respectively). Each dot represents a single donor (*n* = 4). **(D)** CD4^+^ T cells were stained with proliferation dye prior to stimulation with mDC, differentiated from the same donor used in primary stimulation, in presence or absence of autologous T(DC^IL−10^), or T(DC^IL−10−LPS^), or T(DC^IL−10−POLI^) cells at 1:1 ratio. Percentage of proliferated cells at the end of the culture (left) was calculated by overall proliferation dye dilution. IFN-γ production in cell culture supernatants (right panel) was evaluated by ELISA. Each dot represents a single donor (*n* = 4).

Analysis of the expression of the tolerogenic molecules HLA-G and ILT4 on DC^IL−10^ showed that, among the different stimuli used, TLR3- and TLR4-mediated activation had opposite effects: Poli I:C exposure increased HLA-G and decreased ILT4, while LPS stimulation down-regulated HLA-G and up-regulated ILT4 (Figure 6A, Figure S4). Since HLA-G and ILT4 are involved in DC-10-mediated induction of Tr1 cells (32), we investigated how DC^IL−10^ tolerogenic activity varied upon stimulation with LPS (DC^IL−10−LPS^) and Poli I:C (DC^IL−10−POLI^). In line with the changes in HLA-G levels, the percentage of Tr1 cells induced at the end of T cell differentiation was higher in T cells induced by DC^IL−10−POLI^ and lower in T cells induced by DC^IL−10−LPS^. Despite these differences, all the three DC^IL−10^ populations induced Tr1 cells at a higher efficiency when compared to control DC^GFP^ (Figure 6B). No major differences in proliferative capacity, cytokine profile and suppressive ability were observed among Tr1 cells generated with unstimulated DC^IL−10^, DC^IL−10−LPS^, and DC^IL−10−POLI^, showing that DC^IL−10^ maintained their tolerogenic activity upon pro-inflammatory stimulation (Figures 6C,D). Overall, these data indicate that activated DC^IL−10^ are as powerful as their steady state counterpart in modulating T cell responses and in promoting Tr1 cells *in vitro*, suggesting that activation does not impair the modulatory activity and tolerogenic potential of DC^IL−10^.

### Human DC^IL-10^ Modulate Allogeneic T cell Responses *in vivo*

**Figure 7:**
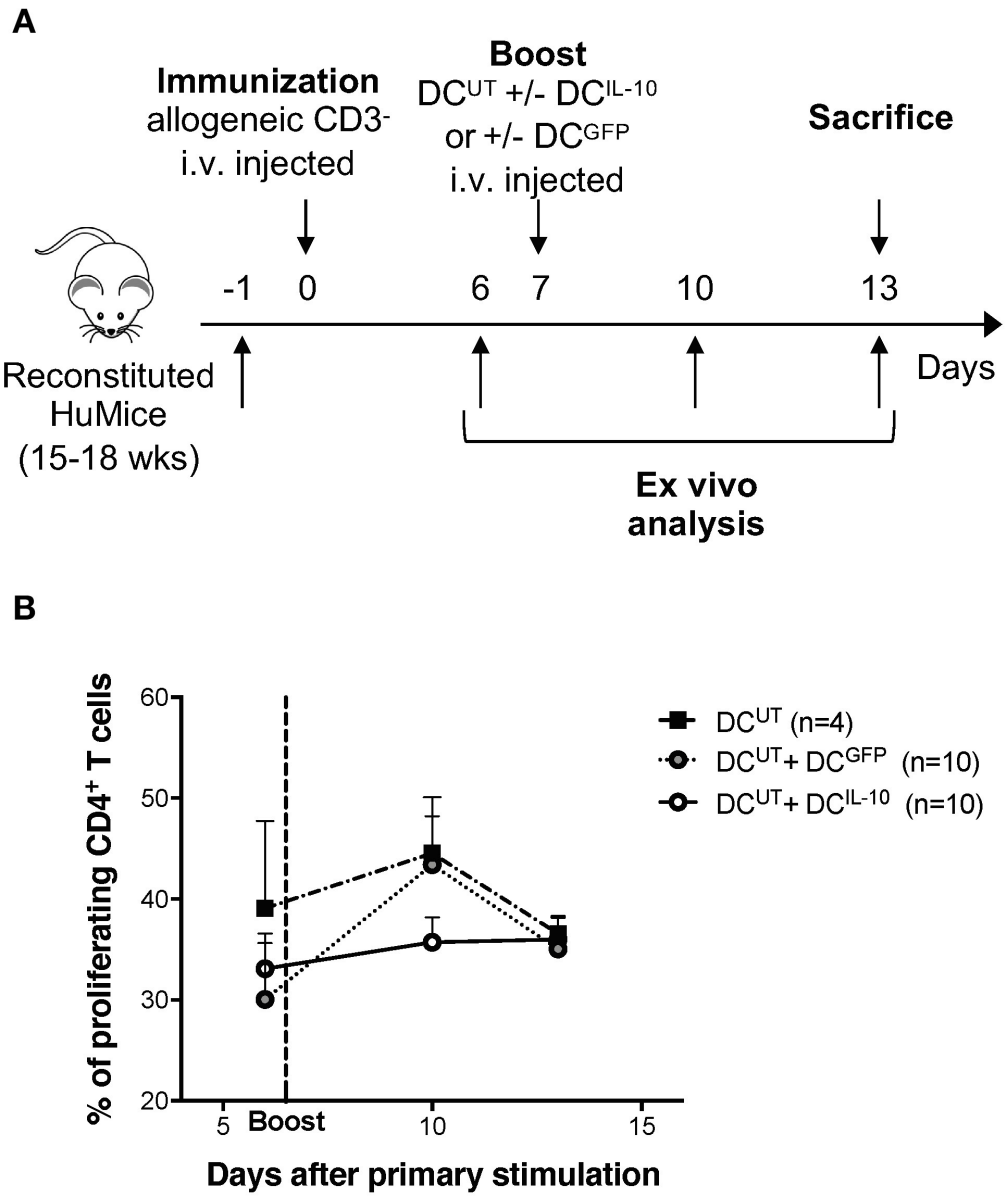
DC^IL−10^ prevent allo-specific T cell reactivation in huMice. NSG mice were transplanted with 10^5^ CD34^+^ human cells. Reconstituted huMice were immunized with i.v. injected irradiated allogeneic CD3^−^ cells (*n* = 24). On day 7, immunized huMice were boosted with autologous un-transduced DC (DC^UT^) alone (*n* = 4), or in combination with DC^IL−10^ (DC^UT^+DC^IL10^) (*n* = 10), or DC^GFP^ (DC^UT^+DC^GFP^) (*n* = 10). **(A)** Experimental design is depicted. **(B)** Peripheral blood CD4^+^ T cell proliferation was assessed by Ki67 staining at day 6, 10, and 13 from immunization. Mean values and SEM are shown.

To assess the modulatory activity of DC^IL−10^ *in vivo* we took advantage of the recently developed protocol for the repopulation of NSG mice with human cord blood CD34^+^ cells. Intra-liver injection of human CD34^+^ cells in sub-lethally irradiated neonate NSG mice allowed efficient engraftment of human CD45^+^ hematopoietic cells in bone marrow (BM) and differentiation of lymphoid (B and T effector, and T regulatory) and myeloid cells in the periphery (28). We immunized reconstituted huMice by injection of irradiated allogeneic human CD3^−^ cells, which act as antigen presenting cells, and provided, after seven days, a second challenge by injecting autologous DC^UT^. To assess the modulatory activity of DC^IL10^, we co-injected DC^IL−10^ (DC^UT^+DC^IL10^) or, as control, DC^GFP^ (DC^UT^+DC^GFP^) (Figure 7A). While treatment with DC^GFP^ induced a boost in CD4^+^ T cell proliferation *in vivo*, as assessed by Ki67 staining of peripheral blood lymphocytes 3 days after the re-challenge, DC^IL−10^ dampened the response induced by allogeneic DC^UT^. In all the three conditions, the immune system returned at steady state after 5 days from the re-challenge, with comparable proliferation levels observed in all mice (Figure 7B).

### Intravenously Injected Murine DC^IL-10^ Engraft Lung and Bone Marrow and Delay Acute Graft-vs. Host Disease

**Figure 8:**
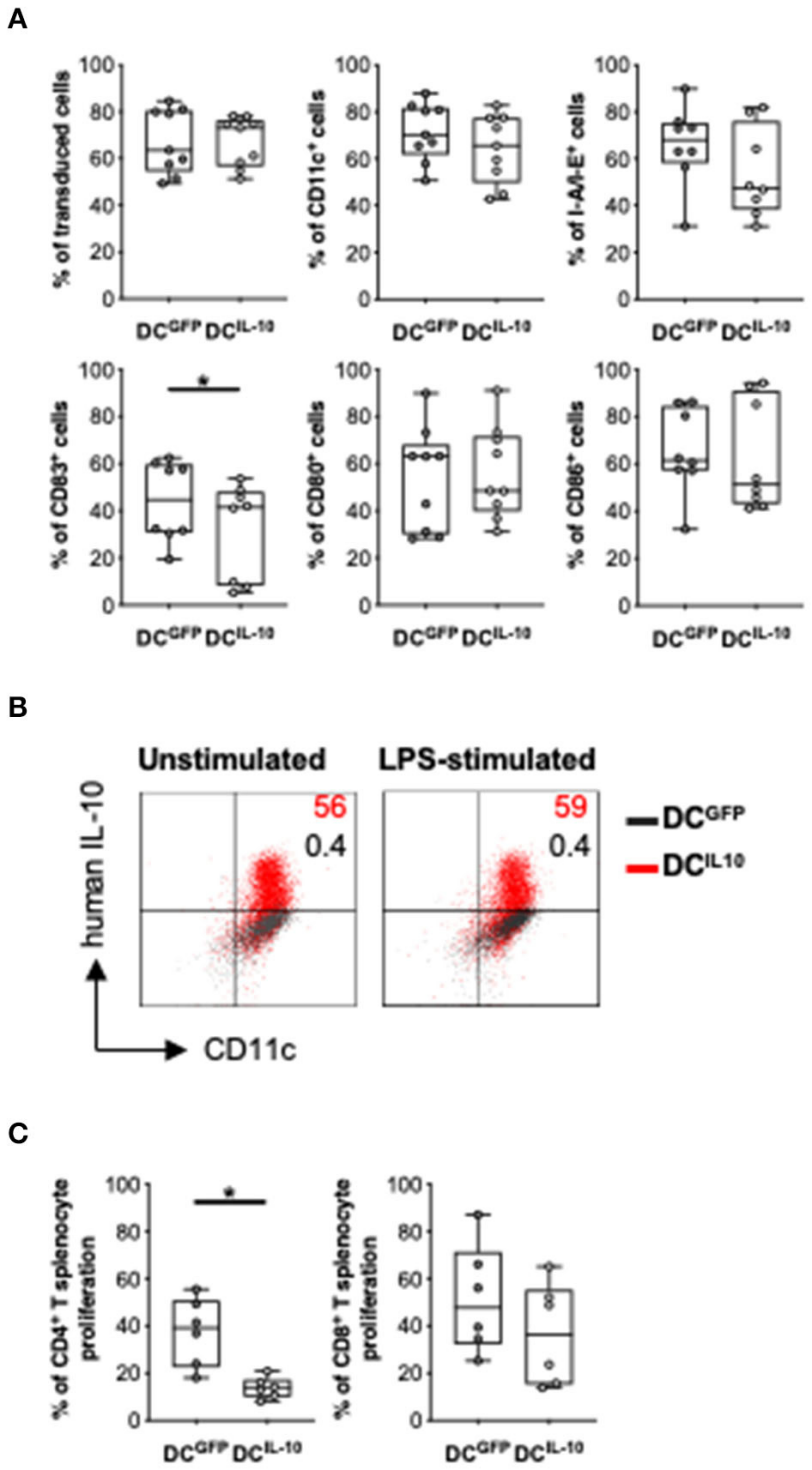
*In vitro* characterization of murine DC^IL−10^. Female Balb/c BM cells were differentiated into DC, transduced at day 2 with LV-GFP (DC^GFP^) or LV-IL-10 (DC^IL−10^), and activated with LPS (200 ng/ml) during the last 2 days of differentiation. **(A)** Transduction efficiency was quantified based on ΔNGFR expression and the expression of the indicated markers was analyzed at day 9 of differentiation by flow cytometry. Each dot represents a single experiment (*n* = 8–9), lines indicate median, while whiskers are minimum and maximum levels. **(B)** DC were plated and left unstimulated or stimulated with LPS (200 ng/ml) for 24 h, with the addition of brefeldin A at 6 h. The expression of human IL-10 was quantified by intracytoplasmic staining. One representative donor out of two is depicted, and percentages of positive cells are indicated. **(C)** Spleen cells from female C57Bl/6 mice were stained with a proliferation dye and stimulated with Balb/c DC^GFP^ and DC^IL−10^ at 1:10 ratio. At day 5, proliferation of CD4^+^ and CD8^+^ T cells was measured by flow cytometry. Each dot represents a single donor (*n* = 6), lines indicate median, while whiskers are minimum and maximum levels. ^*^P ≤ 0.05 (Wilcoxon matched pairs test, two-tailed).

We next investigated the potential therapeutic effect of DC^IL−10^ in a pre-clinical model of acute graft-vs. host disease (GvHD). To do so, we generated and characterized the murine counterpart of the human DC^IL−10^. We transduced Balb/c (H-2d) BM cells with LV-IL-10 and LV-GFP at day 2 during DC differentiation and activated them with LPS in the last 2 days of culture. Murine DC were efficiently transduced with LV-IL-10 and LV-GFP, as demonstrated by an average 67.3 and 67.1% of ΔNGFR^+^ cells, respectively (Figure 8A). In contrast to DC^GFP^, DC^IL−10^ showed lower expression levels of CD83, while the expression of MHC class II, CD80, and CD86 was comparable (Figure 8A). As expected, DC^IL−10^ showed human IL-10 expression at steady state and upon activation, which was not detected in DC^GFP^ (Figure 8B). Similar to human DC^IL−10^, murine DC^IL−10^ promoted a significantly lower proliferative allogeneic T cell response compared to that elicited by DC^GFP^, both in CD4^+^ and CD8^+^ T cells (Figure 8C).

**Figure 9:**
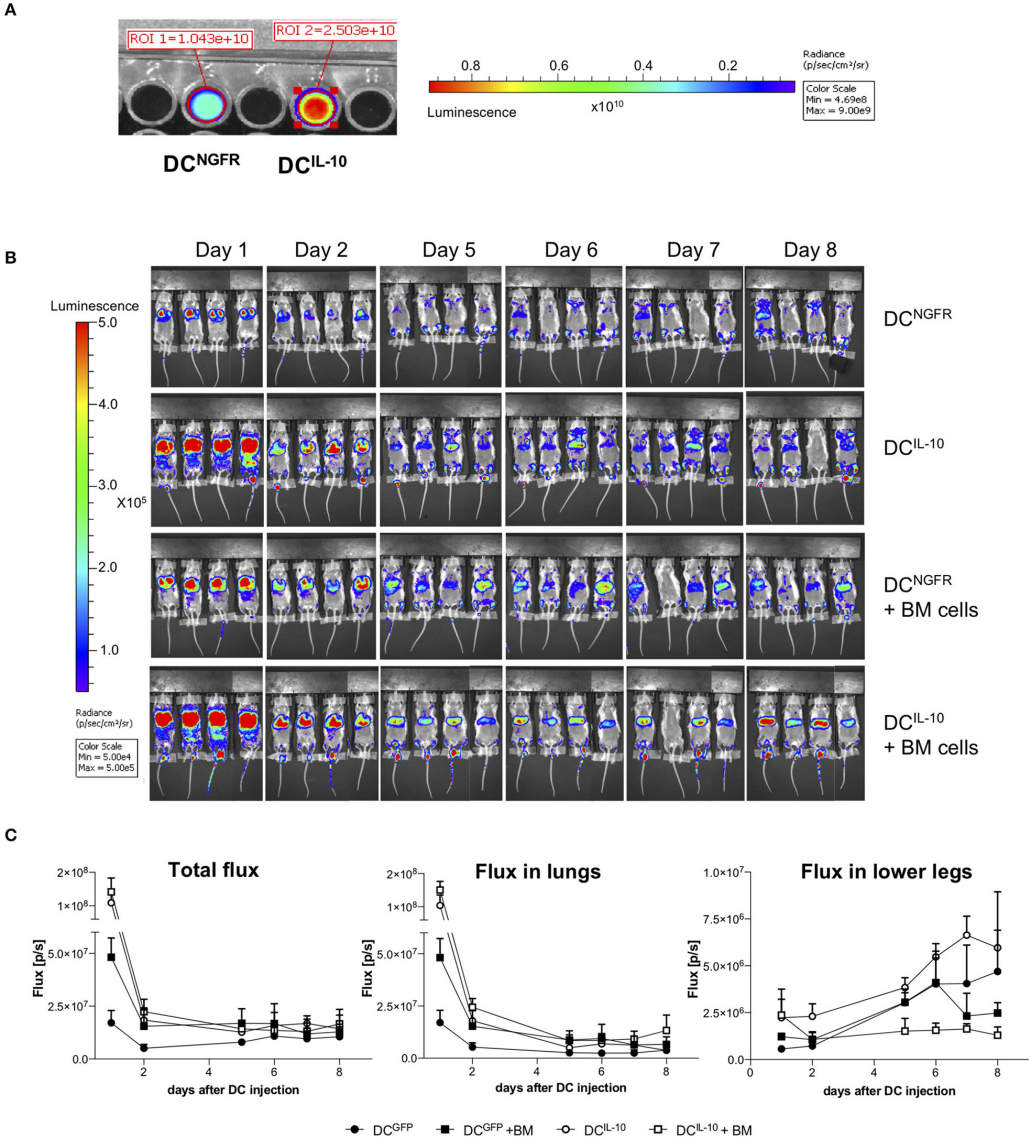
Biodistribution of transduced DC. Female Balb/c BM cells were transduced at day 2 during DC differentiation with bdLV encoding for luciferase and IL-10 (DC^IL−10^) or ΔNGFR (DC^NGFR^). **(A)** Transduction efficiency was evaluated adding luciferin to a well with 2 × 10^5^ DCs and reading the bioluminescent signal. **(B,C)** Female Balb/c mice were lethally irradiated and intravenously injected with luciferase-transduced DC^NGFR^ or DC^IL−10^ (2 × 10^6^ cells/mouse) alone or in combination with autologous BM cells (10^7^ cells/mouse) (*n* = 4 mice for group). DC localization was monitored with bioluminescence imaging (BLI) at the indicated time points. Single mice scanning **(B)** and quantification of BLI at the indicated time points **(C)** are shown.

To study murine DC^IL−10^ biodistribution, we transduced BM cells during DC differentiation with a bdLV encoding for luciferase and IL-10 (DC^IL−10^) or ΔNGFR (DC^NGFR^). DC^IL−10^ and control DC^NGFR^ were then intravenously injected alone or in combination with autologous BM cells in lethally irradiated female Balb/c mice. The difference in the 1st day signals between DC^IL−10^- and DC^NGFR^-injected mice was very likely due to a higher transduction of DC^IL−10^ (Figure 9A). In all groups we observed a consistent decrease in total flux in the first 2 days after DC injection, and both cell types localized in the lung (Figure 9B). Between day 2 and 5 lung signal dropped, and increased signal was registered in the legs of all mice (Figure 9C). When BM cells were co-injected, a different behavior was observed between DC^IL−10^ and DC^NGFR^. DC^NGFR^ migrated to the legs with the same kinetic irrespectively of the presence or absence of BM cells, at least until day 6. Conversely, migration of DC^IL−10^ to the bones is limited by the presence of BM cells, while when mice are not replenished with BM cells it is increased, even at higher rate compared to DC^NGFR^, probably due to an enhanced recruitment induced by the aplastic/hypoplastic bone marrow upon irradiation.

**Figure 10:**
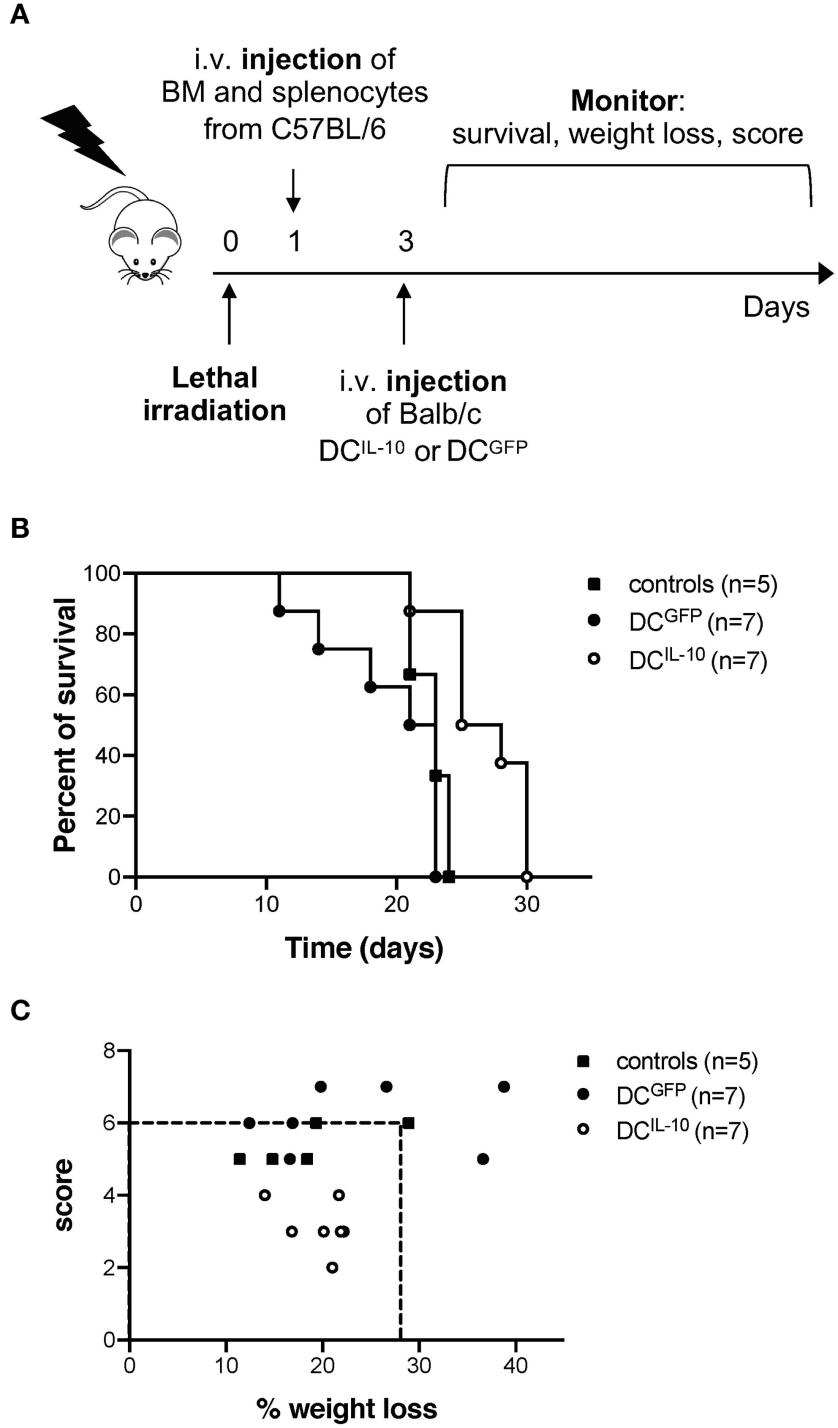
Adoptive transfer of DC^IL−10^ delays acute graft-vs. host disease. Female Balb/c mice were lethally irradiated and then intravenously injected with 10^7^ BM cells and 5 × 10^6^ splenocytes from female C57Bl/6 mice the day after. On day 3, mice were left untreated (*n* = 5) or were adoptively transferred with 2 × 10^6^ DC^GFP^ (*n* = 7), or 2 × 10^6^ DC^IL−10^ (*n* = 7). Survival, weight loss and score of mice was monitored every other day. **(A)** Experimental design is depicted. **(B)** Survival of mice was observed over time. One representative experiment out of three is shown. **(C)** The score (fur, hunch, skin lesion, mobility) and the weight loss of mice at day 25 is represented, dashed lines represent the criteria for euthanasia (score >6 or > 25% of weight loss). One representative experiment out of three is shown.

Finally, we evaluated the potential therapeutic effect of DC^IL−10^ in GvHD. Lethally irradiated female Balb/c recipients were transplanted with BM cells and splenocytes isolated from female C57Bl/6 mice, and 2 days after transplantation mice received Balb/c-derived DC^IL−10^ or DC^GFP^, or were left untreated as control (Figure 10A). This experimental setting induced a strong GvHD, since all mice of the control group died within 24 days (Figure 10B). Administration of Balb/c DC^IL−10^ improved the survival time of mice compared to both control groups (50% survival at day 24), while Balb/c DC^GFP^ treatment enhanced GvHD lethality (Figure 10B). Interestingly, treatment with DC^IL−10^ clearly modulated not only the survival but also the severity of GvHD phenotype in mice, compare to both untreated and DC^GFP^-treated mice groups, as shown by weight loss and score assessment at day 24 (Figure 10C). These results support the potential clinical application of DC^IL−10^ as cell-based approach to control T cell responses in allogeneic transplantation setting.

## Discussion

We designed a powerful method to genetically engineer monocyte-derived DC using bdLV to over-express IL-10 in combination with a marker gene, allowing *in vitro* selection, and *in vivo* tracking of DC^IL−10^. DC^IL−10^ display a strong tolerogenic profile since they: secrete supra-physiological levels of IL-10 at steady state and upon activation, but no pro-inflammatory IL-12; modulate CD4^+^ and CD8^+^ T cell responses and differentiate alloantigen-specific Tr1 cells *in vitro;* and are phenotypically and functionally stable upon TLR-mediated or cytokine-mediated activation. More importantly, human DC^IL−10^ modulate allogeneic CD4^+^ T cell responses *in vivo* in immunized humanized mice and preliminary data showed that murine DC^IL−10^, which share phenotypic and functional characteristics with human DC^IL−10^, delay mice mortality in a model of acute GvHD, dampening the clinical signs of the pathology.

We applied, for the first time, the genetic modification of human monocyte-derived DC with bdLV-encoding for IL-10 and ΔNGFR, to obtain IL-10 over-expressing tolDC. IL-10 transduction has been exploited to generate murine bone marrow-derived IL-10-producing tolDC (34), and used to promote antigen-specific tolerance *in vivo* via Treg induction (35, 36), or to differentiate IL-10-producing Tr1 cells *in vitro* suitable for cell-based approaches in preclinical models of GvHD (37). Human DC^IL−10^ differ from IL-10-treated human monocyte-derived immature and mature DC that express reduced levels of HLA-DR and costimulatory molecules, leading to decreased ability to activate allogeneic T cells (38–40). Indeed, human DC^IL−10^ express HLA-DR, CD83 and CD86 molecules at higher levels compared to control DC, but despite their mature phenotype, they induce suppressive allogeneic T cells *in vitro* and modulate allogeneic T cell responses *in vivo*. DC^IL−10^ are phenotypically and functionally super-imposable to DC-10, which, although differentiated in the presence of IL-10, express HLA-DR and costimulatory molecules at higher levels compared to control DC (32), and are stable upon TLR-activation (33).

Like for DC-10, the ability of DC^IL−10^ to induce Tr1 cells *in vitro* relies not only on the IL-10 secretion, but also on the expression of the tolerogenic molecules HLA-G and ILT4 (32, 41). Their expression is modulated by TLR-mediated stimulation, but with opposite effects: bacterial derived antigens (e.g., LPS and Listeria), decrease HLA-G and up-regulate ILT4, while viral stimuli, such as Poli I:C and CpG, increase HLA-G and down-regulate ILT4. These variations influence the proportion of Tr1 cells induced by DC^IL−10^, but they do not affect the anergic and suppressive phenotype of the generated Tr1 cells, indicating that the high levels of IL-10 secreted by activated DC^IL−10^ compensate for the alteration in the signaling mediated by HLA-G or ILT4. This conclusion is in line with data obtained with tolDC generated in the presence of G-CSF (G-DC), that although express ILT4 and HLA-G at levels comparable to DC-10, do not promote the differentiation of suppressive Tr1 cells (42), because G-DC produce significantly lower levels of IL-10 compared to DC-10 (32).

The ability of tolDC to generate long-term tolerance to the transplant has been proposed as an alternative to current pharmacological approaches used to avoid graft rejection or to prevent GvHD based on general immunosuppression, which could lead to an impairment of the immune system, increasing the risk of infection and cancer (43–46). One of the problems, in the application of tolDC as cell therapy is the stability of the product and the maintenance of their tolerogenic properties *in vivo*. Here, we showed that *in vitro* activation does not strongly affect significantly DC^IL−10^ phenotype or cytokine profile and, despite alteration of the tolerogenic molecule HLA-G and ILT4, their ability to induce Tr1 cells. More interestingly, despite DC^IL−10^ administered in the GvHD model were activated with LPS, they exert their modulatory functions, prolonging the survival of treated mice, and dampening the clinical signs of GvHD in the experiments showed. Systemic administration of tolDC generated with different pharmacological manipulations has been previously shown to modulate GvHD in murine models (47–49). However, different schedule of tolD administration and doses were tested, making comparison difficult. Nevertheless, we cannot exclude that multiple DC^IL−10^ administration, as demonstrated with tolDC manipulated with HDAC inhibitors (49), might results in further improvement of mice survival.

Further preclinical studies are warranted to optimize the protocol for DC^IL−10^ administration, but preliminary data presented here suggest DC^IL−10^ represent a promising cell product for clinical applications: they are stable cells with suppressive functions and as dendritic cells, they have a limited life span *in vivo*, restraining the long-lasting impact on immunity against infections and malignancies. DC^IL−10^ induce allo-specific hypo-responsiveness in effector CD4^+^ and CD8^+^ T cells and, through the stable over-expression of IL-10, we hypothesize they generate a local microenvironment enriched in IL-10 that modulate not only T cells, but also myeloid and innate cells, thus sustaining long-term tolerance.

## Data Availability Statement

The raw data supporting the conclusions of this article will be made available by the authors, without undue reservation.

## Ethics Statement

The animal study was reviewed and approved by the OSR Institutional Animal Care and Use Committee (IACUC protocol #488, #632, and #748), following the 3R principles (replacement, reduction, and refinement), and the Decreto Legislativo #116 dated January 27th, 1992, from the Italian Parliament.

## Author Contributions

MC performed the experiments, collected, analyzed, and interpreted data, performed statistical analysis, and wrote the manuscript. LP, MF, GAm, and GAn performed some of the experiments. FS conceived, performed, and analyzed humanized mice experiments. LP, FR, LC, and GAn performed the *in vivo* experiments. LC produced lentiviral vectors. AK-R contributed Vpx plasmid reagent. SG conceived the scientific idea, supervised the project, intertreted data and wrote the manuscript.

## Conflict of Interest

The authors declare that the research was conducted in the absence of any commercial or financial relationships that could be construed as a potential conflict of interest.
