# Corrigendum: Generation of Powerful Human Tolerogenic Dendritic Cells by Lentiviral-Mediated IL-10 Gene Transfer

**DOI:** 10.3389/fimmu.2021.672701

**Published:** 2021-03-22

**Authors:** Michela Comi, Giada Amodio, Laura Passeri, Marta Fortunato, Francesca Romana Santoni de Sio, Grazia Andolfi, Anna Kajaste-Rudnitski, Fabio Russo, Luca Cesana, Silvia Gregori

**Affiliations:** San Raffaele Telethon Institute for Gene Therapy (SR-TIGET), San Raffaele Scientific Institute (IRCCS), Milan, Italy

**Keywords:** dendritic cells, IL-10, cell therapy, immune tolerance, allogeneic transplantation

In the original article, there was a mistake: a duplication of one dot plot in **Figure 8B** as published. The corrected **Figure 8** appears below.

**Figure 8:**
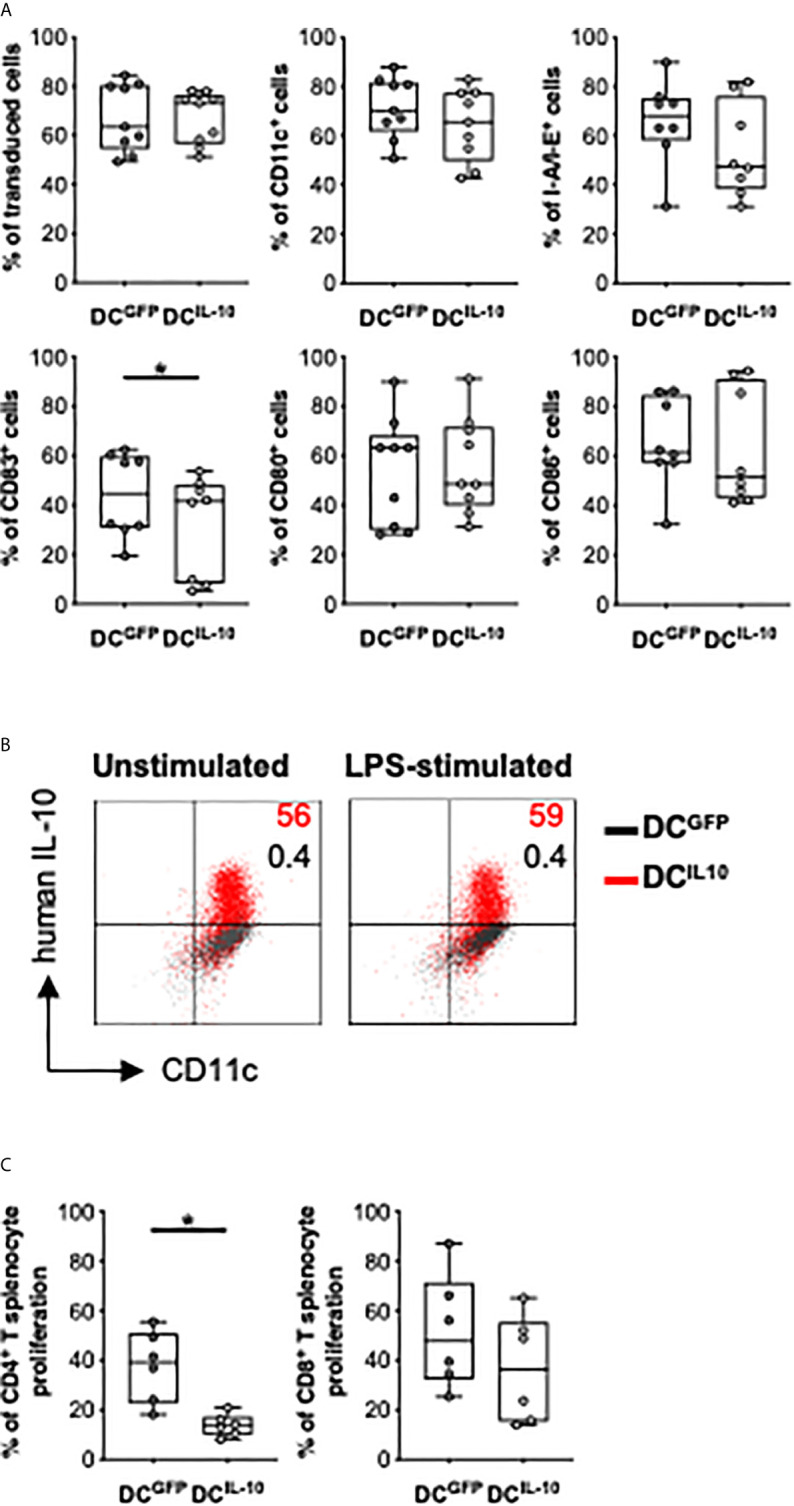
*In vitro* characterization of murine DC^IL-10^. Female Balb/c BM cells were differentiated into DC, transduced at day 2 with LV-GFP (DC^GFP^) or LV-IL-10 (DC^IL-10^), and activated with LPS (200 ng/ml) during the last 2 days of differentiation. **(A)** Transduction efficiency was quantified based on ∆NGFR expression and the expression of the indicated markers was analyzed at day 9 of differentiation by flow cytometry. Each dot represents a single experiment (n = 8-9), lines indicate median, while whiskers are minimum and maximum levels. **(B)** DC were plated and left unstimulated or stimulated with LPS (200 ng/ml) for 24 h, with the addition of brefeldin A at 6 h. The expression of human IL-10 was quantified by intracytoplasmic staining. One representative donor out of two is depicted, and percentages of positive cells are indicated. **(C)** Spleen cells from female C57Bl/6 mice were stained with a proliferation dye and stimulated with Balb/c DC^GFP^ and DC^IL-10^ at 1:10 ratio. At day 5, proliferation of CD4^+^ and CD8^+^ T cells was measured by flow cytometry. Each dot represents a single donor (n = 6), lines indicate median, while whiskers are minimum and maximum levels. *P ≤ 0.05 (Wilcoxon matched pairs test, two-tailed).

The authors apologize for this error and state that this does not change the scientific conclusions of the article in any way. The original article has been updated.

