# Supplementary material for: Generation of Powerful Human Tolerogenic Dendritic Cells by Lentiviral-Mediated IL-10 Gene Transfer
Source: Front Immunol. 2020 Jun 30;11:1260. doi: 10.3389/fimmu.2020.01260 (PMC7338371; doi:10.3389/fimmu.2020.01260)
Supplement: Supplementary file 1 [file Image_1.pdf]

Generation of powerful human tolerogenic dendritic cells by lentiviral-mediated IL-10 gene transfer

Michela Comi[1], Giada Amodio[1], Laura Passeri[1], Marta Fortunato[1], Francesca Romana Santoni de Sio[1], Grazia Andolfi[1], Anna Kajaste-Rudnitski[1], Fabio Russo[1], Luca Cesana[1], and Silvia Gregori[1,*]

[1]San Raffaele Telethon Institute for Gene Therapy (SR-TIGET), San Raffaele Scientific Institute (IRCCS) Milan, Italy

[*]**Correspondence**:
Silvia Gregori,
San Raffaele Telethon Institute for Gene Therapy (SR-TIGET),
IRCCS San Raffaele Scientific Institute.
Via Olgettina, 58, 20132 Milan, Italy.
Phone: +39 0226434894;
Fax: +39 0226434668;
E-mail: gregori.silvia@hsr.it

Keywords: dendritic cells, IL-10, cell therapy, immune tolerance, allogeneic transplantation

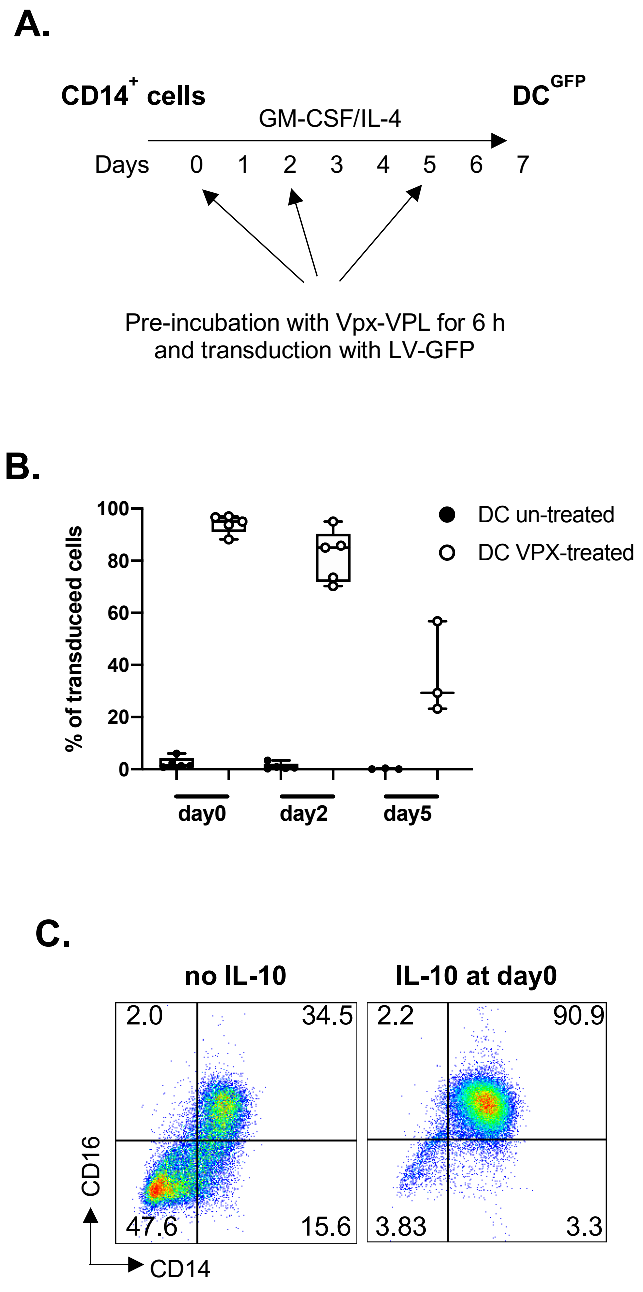

Comi et al., Supplementary Fig.1

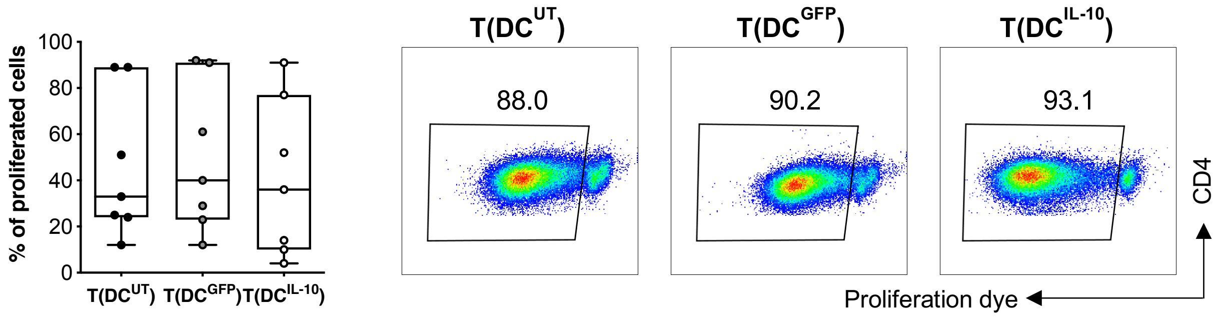

Comi et al., Supplementary Fig.2

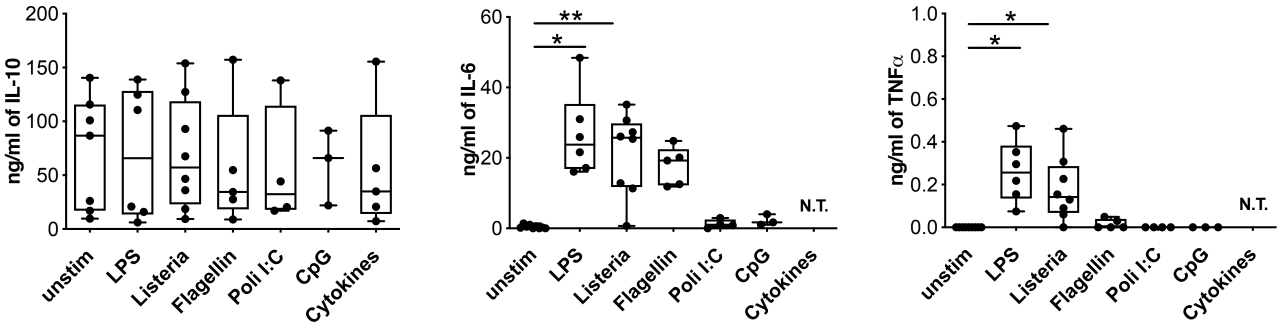

Comi et al., Supplementary Fig.3

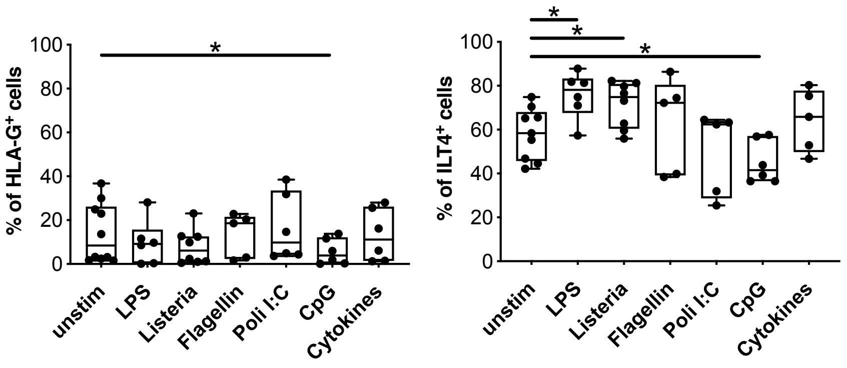

Comi et al., Supplementary Fig. 4

Supplementary Fig.1. Establishment of a protocol to genetically engineer human DC. A-B. $CD14^+$ cells isolated from peripheral blood of healthy subjects (n=3-5) were pre-treated with Vpx-VLP for 6 hours and transduced with LV-GFP (DC^{GFP}) (DC Vpx-treated) at day 0, day 2 and day 5 during DC differentiation. As control, DC transduced with LV-GFP without Vpx-VLP pre-treatment (DC un-treated) were differentiated from the same donors. Protocol of LV-mediated transduction of monocyte-derived DC is depicted (**A**). On day 7, the transduction efficiency was evaluated as the expression of ΔNGFR by flow cytometry. Each dot represents a single donor, lines indicate median, while whiskers are minimum and maximum levels (**B**). **C.** $CD14^+$ cells isolated from peripheral blood of healthy subject were pre-treated with Vpx-VLP for 6 hours at day 0, transduced with LV-IL-10 in the absence (no IL-10) or presence of IL-10 (IL-10 on day 0), and then differentiated to DC in the presence of IL-4 and GM-CSF. Dot plots from one representative donor out of three are presented, percentages of positive cells are indicated.

Supplementary Fig.2. DC^{IL-10} induction of $CD4^+$ T cell anergy is alloantigen-specific. Allogeneic $CD4^+$ T cells were isolated from peripheral blood and cultured with DC^{UT} [T(DC^{UT})], DC^{GFP} [T(DC^{GFP})] or DC^{IL-10} [T(DC^{IL-10})] at 10:1 ratio. After 10 days, $CD4^+$ T cells were purified by positive selection, stained with proliferation dye and re-stimulated with mDC differentiated from an unrelated donor. Proliferation was assessed by proliferation dye dilution after 5 days. Each dot represents a single donor (n=7), lines indicate median, while whiskers are minimum and maximum levels (left panel). Dot plots from one representative donor are presented, percentages of gated cells are indicated (right panel).

Supplementary Fig.3. Upon activation, DC^{IL-10} maintain their cytokine production profile. At the end of differentiation, 10^5 DC^{IL-10} were plated in 200 μl and left unstimulated or activated with LPS, Heat Killed Listeria Monocytogenes, Flagellin S. typhimurium, Poli I:C, ODN2006 (CpG) or a mix of cytokines (IL-1β, TNF-α and IL-6). After 24 hours, the secretion levels of the indicated cytokines were evaluated in culture supernatants by ELISA. Each dot represents a single donor (n=5), lines indicate median, while whiskers are minimum and maximum levels. N.T. = not tested. *P≤0.05 (Wilcoxon matched pairs test, two-tailed).

Supplementary Fig.4. Upon activation, DC^{IL-10} maintain their phenotype. At the end of differentiation, DC^{IL-10} were activated with LPS, Heat Killed Listeria Monocytogenes, Flagellin S. typhimurium, Poli I:C, ODN2006 (CpG) or a mix of cytokines (IL-1β, TNF-α and IL-6). After 24 hours, the expression of the HLA-G and ILT-4 was evaluated by flow cytometry (n=5-10). Each dot represents a single donor, lines indicate median, while whiskers are minimum and maximum levels. *$P \leq 0.05$ (Wilcoxon matched pairs test, two-tailed).
